# Roles of Sorcin in Drug Resistance in Cancer: One Protein, Many Mechanisms, for a Novel Potential Anticancer Drug Target

**DOI:** 10.3390/cancers12040887

**Published:** 2020-04-06

**Authors:** Theo Battista, Annarita Fiorillo, Valerio Chiarini, Ilaria Genovese, Andrea Ilari, Gianni Colotti

**Affiliations:** 1 Department of Biochemical Sciences, Sapienza University, P.le A.Moro 5, 00185 Rome, Italy; theo.battista@uniroma1.it (T.B.); annarita.fiorillo@uniroma1.it (A.F.); 2 Doctoral Programme in Integrative Life Science, Institute of Biotechnology, University of Helsinki, 00014 Helsinki, Finland; valerio.chiarini@helsinki.fi; 3 Department of Medical Sciences, Laboratory for Technologies of Advanced Therapies, University of Ferrara, 44121 Ferrara, Italy; ilaria.genovese@unife.it; 4 Institute of Molecular Biology and Pathology, Italian National Research Council, Istituto di Biologia e Patologia Molecolari, Consiglio Nazionale delle Ricerche (IBPM-CNR), c/o Department of Biochemical Sciences, Sapienza University, P.le A.Moro 5, 00185 Rome, Italy

**Keywords:** sorcin, ABCB1, multidrug resistance, cancers, chemotherapeutic drugs, calcium, endoplasmic reticulum

## Abstract

The development of drug resistance is one of the main causes of failure in anti-cancer treatments. Tumor cells adopt many strategies to counteract the action of chemotherapeutic agents, e.g., enhanced DNA damage repair, inactivation of apoptotic pathways, alteration of drug targets, drug inactivation, and overexpression of ABC (Adenosine triphosphate-binding cassette, or ATP-binding cassette) transporters. These are broad substrate-specificity ATP-dependent efflux pumps able to export toxins or drugs out of cells; for instance, ABCB1 (MDR1, or P-glycoprotein 1), overexpressed in most cancer cells, confers them multidrug resistance (MDR). The gene coding for sorcin (SOluble Resistance-related Calcium-binding proteIN) is highly conserved among mammals and is located in the same chromosomal locus and amplicon as the ABC transporters ABCB1 and ABCB4, both in human and rodent genomes (two variants of ABCB1, i.e., ABCB1a and ABCB1b, are in rodent amplicon). Sorcin was initially characterized as a soluble protein overexpressed in multidrug (MD) resistant cells and named “resistance-related” because of its co-amplification with ABCB1. Although for years sorcin overexpression was thought to be only a by-product of the co-amplification with ABC transporter genes, many papers have recently demonstrated that sorcin plays an important part in MDR, indicating a possible role of sorcin as an oncoprotein. The present review illustrates sorcin roles in the generation of MDR via many mechanisms and points to sorcin as a novel potential target of different anticancer molecules.

## 1. Introduction

Sorcin (SOluble Resistance-related Calcium-binding proteIN) is one of the most expressed calcium-binding proteins in many tissues (source Protein Abundance Database, PaxDb, https://pax-db.org/). Although its most characterized function concerns the regulation of cardiac contractile activity, a significant role has emerged in the context of cancer and, especially, in multidrug resistance (MDR). In fact, sorcin is overexpressed in many human cancers, as lymphomas, leukemias, gastric, breast, lung, nasopharyngeal, ovarian tumors, adenocarcinoma, glioblastoma, astrocytoma, oligodendroglioma, and multidrug (MD)-resistant tumors, with respect to normal tissues (for a review, [1,2]).

In leukemia patients, sorcin expression levels inversely correlate with response to chemotherapies and with overall prognosis. Sorcin is overexpressed in cell lines resistant to chemotherapeutic drugs and significantly upregulated in the doxorubicin-induced MD-resistant leukemia K562/A02 cell line with respect to its parent cells. Sorcin overexpression by gene transfection: (i) increased drug resistance to a variety of chemotherapeutic agents (e.g., doxorubicin, etoposide, homoharringtonine, and vincristine) in K562 cells; and (ii) determined drug resistance (to vincristine, adriamycin, taxol, and 5-fluorouracil) in SGC7901 cells, ovarian and breast cancer. On the other hand, several recent studies have demonstrated that inhibition of sorcin expression by RNA interference led to a reversal of drug resistance in a number of cell lines.

**Figure 1:**
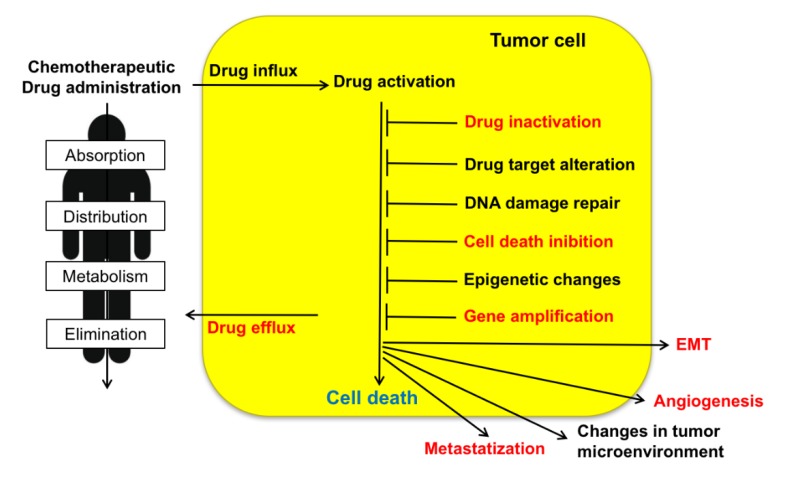
Upon administration of chemotherapeutic drugs, intrinsic or extrinsic factors determine multidrug resistance (MDR). These include absorption, distribution, metabolism, and elimination (ADME), drug influx, drug efflux, drug activation and inactivation, drug target alteration, DNA damage repair, cell death (in particular apoptosis) inhibition, epigenetic effects, epithelial-to-mesenchymal transition (EMT), changes in tumor environment, angiogenesis, metastasis. Sorcin participates in several of such MDR mechanisms (indicated in red, see text).

Resistance to chemotherapeutic treatments is one of the main challenges in the fight against cancer. Tumor cells can adopt several strategies to evade death induced by chemotherapeutic agents. These include changes in apoptotic pathways, increased DNA damage repair, drug inactivation, alteration of drug targets, and increased expression of ABC (ATP-binding cassette) transporters (Figure 1) [3]. As illustrated in the present review, sorcin participates in many of such strategies (Table 1). Taken together, the above data indicate that sorcin has a significant and general role in MDR so that it can be a useful marker of MDR and may represent a therapeutic target for reversing tumor MDR. 

## 2. Role and Mode of Action of Sorcin in Physiological and Pathological Processes

### 2.1. Sorcin Structure and Calcium-dependent Activation

Sorcin (SOluble Resistance-related Calcium-binding proteIN) was labeled “resistance-related” since it was found co-amplified with ABCB1 in MD-resistant cells [73]. The gene coding for sorcin (*SRI*) is about 21.9 kb-long and is located in chromosome 7 (region 7q21). At least four different Sorcin isoforms are transcribed in human, i.e., isoforms A (a 15 kb-transcript, with 8 exons and 7 introns, translated into a 22-kDa, 198-residues long isoform), B, C, and D (translated into shorter, 19-kDa, isoforms, lacking part of the N-terminal domain and/or the last amino acids of the C-terminal domain); the 22-kDa isoform A is the most studied sorcin isoform, although some studies refer to 19-kDa forms of the protein. A sorcin-like pseudogene (SRIL) is located in chromosome 4 [2].

**Figure 2:**
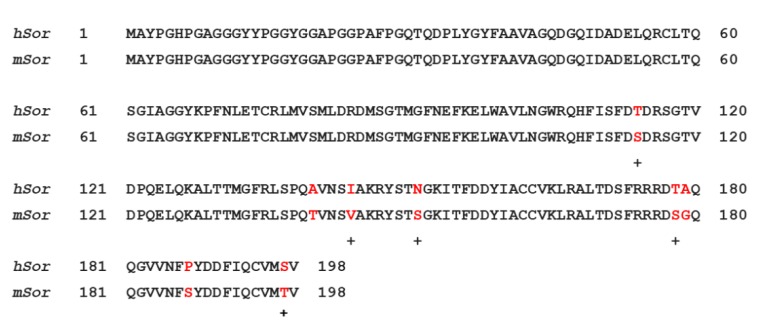

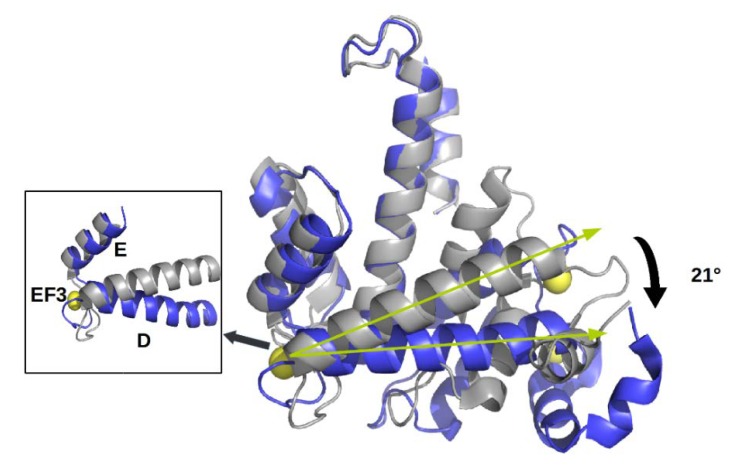
Upper panel. Alignment between human sorcin (*hSor*) and mouse sorcin (*mSor*). The variant residues are indicated in red. The “+” indicates residues with similar characteristics. Lower panel. The X-ray crystal structure of human sorcin in the apo form (gray) and in the calcium-bound form (blue; calcium ions are represented by yellow spheres). Upon calcium binding, sorcin activation occurs, with a transition from a closed to an open structure (see also detail of the EF3 hand), involving a movement of the long D-helix of 21°.

Sorcin is evolutionarily rather recent, being present in vertebrates, and more generally in metazoans. Sorcin sequence is highly conserved among species, e.g., human and mouse sorcin differ only by eight residues (T114S, A140T, I144V, N151S, T178S, A179G, P187S, S197T) (Figure 2, upper panel) among which three (in both human and mouse sorcin) are phosphorylatable serine and threonine residues of the C-terminal domain, possibly indicating species-specific phosphorylation-dependent sorcin regulation.

From a structural viewpoint, sorcin belongs to the small penta-EF-hand (PEF) family, which also comprises calpains, grancalcin, PDCD6, and peflin [79]. EF-hands are structural helix–loop–helix motifs, with a 12-residue interhelical sequence, able to bind Ca^2+^ with high affinity (with a pentagonal bipyramidal symmetry): calcium binding to proteins acts as a signal in a variety of cellular processes. Sorcin is a homodimer in the absence of calcium [4]; each monomer is formed by two domains, i.e., the flexible glycine-rich N-terminal domain (residues 1–32) and the C-terminal Ca^2+^ binding domain (SCBD, residues 33–198) containing with five EF-hands. Usually, Ca^2+^ binding proteins are endowed with an even number of EF-hands, both structurally and functionally coupled. In sorcin, EF-hands are coupled via short two-stranded β-sheets, such that EF1-EF2 and EF3-EF4 pairs are formed; EF5, although uncoupled in sorcin monomers, pairs with another EF5 hand (belonging to the second monomer) in dimeric sorcin, thereby forming part of the dimer interface [29,30,36].

**Figure 3:**
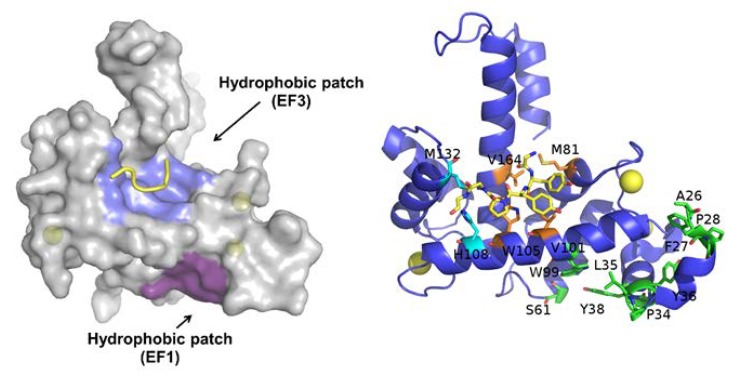
Ca^2+^-bound sorcin in complex with a peptide belonging to the N-terminal domain. Left: Upon calcium binding to sorcin, two hydrophobic patches are exposed to the solvent and likely mediate target binding. One patch (violet) arises from the opening of EF1, the other (blue) from EF3. The peptide belonging to the sorcin N-terminal domain is shown in yellow. Right: detail of the residues involved in the exposure of the hydrophobic surfaces upon calcium binding to sorcin, belonging to the A-helix and EF1 hand (green), and to the C-helix, D-helix, EF4 loop, and G-helix (orange and cyan). The peptide belonging to the sorcin N-terminal domain (in yellow) and the residues interacting with it are represented as sticks.

Sorcin activation is calcium-dependent. Upon Ca^2+^ binding to EF1-3 hands, sorcin undergoes a large conformational change [4,5,80] (Figure 2) that involves a 21° movement of the long D-helix that joins the EF1-EF2 subdomain to EF3, opens EF1, and exposes hydrophobic surfaces in the EF1-EF3 region (Figure 3). This allows sorcin to aggregate in the absence of protein targets or to bind and regulate several proteins in a Ca^2+^-dependent manner [4,5,6,7,8,29,80]. Peptide phage display experiments identified two consensus sequences by which target proteins bind sorcin upon Ca^2+^ binding, i.e., a Φ/Gly/Met-Φ/Gly/Met-x-P motif, where Φ is an aromatic residue (Trp, Tyr, or Phe) and x is any amino acid, and an acidic-Φ motif [29]. The Φ/Gly/Met-Φ/Gly/Met-x-P motif is consistent with the sequence of sorcin N-terminal peptide, found in the hydrophobic pocket exposed in the D helix-EF3 region, which comprises residues Trp105 and His108, possibly the most important residues for interaction with targets (Figure 3) [5,6,29,80]. 

### 2.2. Sorcin Mechanisms of Action: Role in Calcium Homeostasis, ER Stress, and Apoptosis

Sorcin is highly expressed in many tissues: it is among the top 3% expressed proteins of the human proteome and one of the most expressed Ca^2+^ binding proteins (source PaxDb, https://pax-db.org/), and has an essential role in calcium homeostasis [22]. 

Sorcin participates in several processes in the cell and is essential for mitotic progression and cytokinesis: sorcin silencing determines important problems in mitosis and cytokinesis, increases the number of polynucleated rounded cells, and results in blockage of the cell cycle in G2/M, apoptosis and cell death [22]. 

In 3T3-L1 fibroblasts, sorcin localizes dynamically during cell cycle progression. In interphase, sorcin is in the nucleus (where it is distributed in a speckled fashion and excluded from the nucleoli), in the plasma membrane, in the endoplasmic reticulum (ER) and in ER-derived vesicles localized along the microtubules. These vesicles are positive to Ryanodine Receptors (RyRs), sarcoplasmic/endoplasmic (SR/ER) reticulum Ca^2+^-ATPase (SERCA), Rab10, and calreticulin. At the beginning of mitosis, i.e., in prophase and upon disruption of the nuclear envelope, sorcin accumulates in the apical zone of the mitotic spindle, while in metaphase, upon chromosome separation, most sorcin accumulates in the central region of the spindle. In the early telophase, sorcin localizes to the cleavage furrow, while in late telophase, most sorcin moves back to the reforming nuclei, but a significant part flanks the central region of the midbody [22].

**Figure 4:**
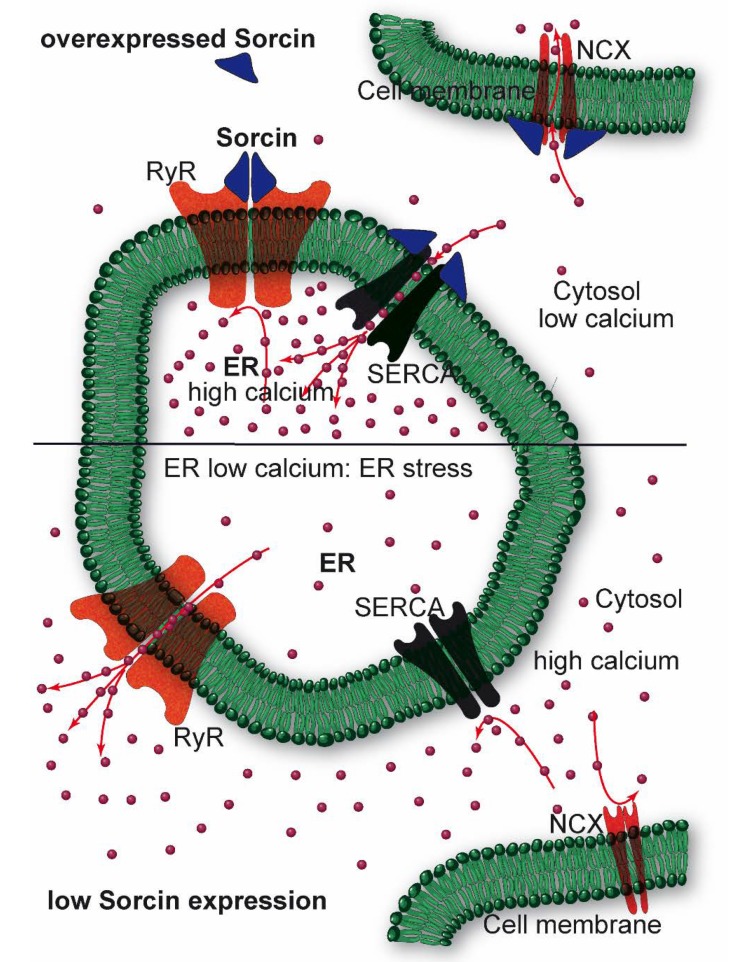
Sorcin inhibits Ryanodine Receptors (RyRs) and activates sarco/endoplasmic reticulum Ca^2+−^ATPase (SERCA) and Na^+^/Ca^2+^ exchanger (NCX), thereby increasing Ca^2+^ load of the endoplasmic reticulum (ER) and decreasing ER stress (**top**). When sorcin expression is low, ER Ca^2+^ load is decreased, thereby increasing ER stress (**bottom**).

Sorcin regulates size and Ca^2+^ content of the ER and ER vesicles, inhibiting RyR, and activating SERCA (Figure 4). Sorcin regulates Ca^2+^ homeostasis in the cells. In the heart, sorcin participates in the regulation of cardiac excitation-contraction coupling, through its critical role in maintaining calcium homeostasis and regulating Ca^2+^ fluxes in the cardiomyocyte.

Cycles of excitation, contraction, and relaxation take place in about 800 ms. The electrical excitation of cardiomyocytes is started by a depolarization wave that opens the voltage-dependent Na^+^ channels of the T-tubules, resulting in membrane depolarization and calcium entry through voltage-operated Ca^2+^ channels. Calcium influx locally increases Ca^2+^ concentration near RyRs, and activates RyR-dependent Ca^2+^ release from the SR, thereby further increasing cytosolic Ca^2+^ concentration; the cation binds to troponin C and triggers cardiac contraction. Relaxation follows rapidly: the RyR channels close, and calcium efflux out of the cytosol takes place through SERCA, which pumps the ion back into the SR, and Na^+^/Ca^2+^ exchanger (NCX) at the plasma membrane (and mitochondria) [81]: cytosolic calcium concentration decreases rapidly, and calcium dissociates from the myofilaments, switching muscle relaxation. 

Upon calcium-dependent activation, sorcin rapidly binds to RyR, inhibiting single-channel activity, thereby attenuating Ca^2+^-induced Ca^2+^ release by SR/ER and decreasing Ca^2+^-triggered membrane depolarization [6,7,9,10]. In addition, sorcin increases SERCA activity, increasing SR/ER calcium load [11] (Figure 4). Further, sorcin increases the activity of the sarcolemmal NCX ^14^, stimulates voltage-dependent inactivation, and slows Ca^2+^-dependent inactivation of the L-type voltage-dependent Ca^2+^ channel (LTCC) [12,13]. Sorcin overexpression enhances cardiac contractility and reverses contractile anomalies of diabetic cardiomyopathy [14,15,16,17], while sorcin KO mice exhibit arrhythmias and sudden death under acute or chronic stress, due to disturbances of Ca^2+^ fluxes [18]. Sorcin is an important player in other cells where excitation-contraction cycles take place, as outer hair cells, that amplify the acoustic signal in the ear [19]. In general, sorcin regulates calcium homeostasis in all types of cell, decreasing free cytosolic Ca^2+^ and increasing ER Ca^2+^ concentration, by binding calcium and by regulating the same channels, pumps, and exchangers, possibly protecting cells from ER stress, dependent on decreased calcium concentration in the organelle (Figure 4).

Besides calcium channels, sorcin interacts in Ca^2+^-dependent fashion with many protein targets, including Polo-like kinase 1 (PLK1), Aurora A and Aurora B kinases, involved in cell cycle regulation. Sorcin physically interacts with PLK1 and induces PLK1 autophosphorylation, regulating kinase activity [22]. Further, PLK1, Ca^2+^-calmodulin dependent kinase II (CaMKII), and cyclic adenosine monophosphate (cAMP)-dependent protein kinase (PKA) phosphorylate sorcin, thus regulating sorcin binding to RyRs and SERCA, and eventually Ca^2+^ homeostasis [22,30,33]. Additionally, sorcin interacts with the calcium-dependent, phospholipid-binding proteins Annexins A7 and A11 [5,6,22,80,82]; in particular, Annexin A11, like sorcin, is needed for midbody organization and for cytokinesis [83].

Sorcin was identified in many types of vesicles, indicating a particularly significant but still puzzling role in the trafficking of various cell types and tissues. In addition to ER-dependent vesicles, sorcin was identified in nanovesicles released in a Ca^2+^-dependent fashion from the erythrocytes and containing Annexin A7 [84]; further, sorcin was identified in many types of exosomes, from B-cells, T-cells, mesenchymal stem cells, breast milk, plasma, red blood cells, seminal plasma, human urine and platelets, thymus, dendritic cells, cerebrospinal fluid, and from many types of cancer cells, such as ovarian cancer, prostate cancer, squamous carcinoma, melanoma, lung cancer, chronic lymphocytic leukemia, colorectal cancer, osteosarcoma, astrocytoma, glioblastoma, and neuroblastoma [72,85,86,87,88,89,90,91,92].

In many tumor cells, sorcin is overexpressed (see below, Section 2.4.). Sorcin-overexpressing vincristine- and daunorubicin-resistant Ehrlich ascites cancer cells have lower cytosolic free Ca^2+^ concentration than the corresponding wild-type cells [23]. In these cells, sorcin silencing increases cytosolic calcium and increases cell death by apoptosis [24], while sorcin overexpression in K562 leukemia cells significantly reduces cytosolic Ca^2+^ levels, thus protecting cells from etoposide-dependent apoptosis, upregulates Bcl-2 and decreases Bax [25]. Since sorcin increases ER Ca^2+^ accumulation, thereby limiting ER stress, it is upregulated in ER stress conditions; conversely, its silencing activates caspase 12, caspase 3, and GRP78/BiP, triggering apoptosis with a mechanism possibly involving the mitochondrial chaperone TRAP1 [26,27].

Taxol-resistant, sorcin-overexpressing A549 non-small-cell lung cancer cells show decreased RyR currents, altered ER calcium homeostasis, possibly increased ER Ca^2+^ reuptake by SERCA and/or increases Ca^2+^ efflux by NCX, and increased Bcl-2 expression [20].

In myeloma cells, sorcin silencing reduces cell proliferation, cell cycle blockage, and apoptosis, and significantly reduces the expression levels (both mRNA and protein) of the xenobiotic pumps ABCB1 and MRP1, of GST-π, Survivin, Bcl-2, Livin, phospho-Src, Cyclin-D1, p21, C-myc, phospho-Akt, and NF-κB, while significantly increasing the expression of p53 and the activity of caspase 3 and caspase 8 [32].

### 2.3. Sorcin Under Cellular Stressing Conditions and in Pathologies

Sorcin is important for glucose tolerance and protects vs. lipotoxicity in vivo: sorcin downregulation occurs under lipotoxic stress conditions, such as high-fat diet and exposure to proinflammatory cytokines or palmitate [28,93], while sorcin overexpression protects against ER stress. Sorcin deletion impairs glucose tolerance and glucose-stimulated insulin secretion (GSIS) in transgenic mice, whereas sorcin overexpression in pancreatic β-cells increases glucose tolerance and enhances GSIS during high-fat diet [28]. Sorcin increases intracellular Ca^2+^ fluxes and ER Ca^2+^ stores, regulates glucose-6-phosphatase catalytic subunit-2 (G6PC2) via nuclear factor of activated T-cells (NFAT) activation, decreases the levels of C/EBP homologous protein (CHOP) and Grp78/BiP, i.e., of ER stress markers and activates the transcriptional activity of the activating transcription factor 6 ATF6 [28]. 

In turn, sorcin silencing activates apoptotic caspase-3 and caspase-12, Bcl-2, Bax, Grp78/BiP, c-fos, c-jun, increases mitochondrial Ca^2+^ concentration and release of cytochrome c [17,22,28]. At low glucose concentrations, sorcin also retains in the cytosol the carbohydrate-responsive element-binding protein ChREBP, i.e., one of the most important mediators of glucotoxicity and regulators of pancreatic β-cell gene expression: sorcin operates as a calcium sensor for glucose-dependent nuclear translocation and for ChREBP-controlled gene activation [35].

High levels of expression in the central nervous system, different in basal and pathological conditions, indicate that sorcin can possibly be a notable player in brain functions and dysfunctions, likely by its capability to modulate calcium homeostasis.

High amounts of sorcin are expressed in the brain (about 5–10 times higher than in the heart): Sorcin is among the most expressed calcium-binding proteins in the amygdala, the hypothalamus, the prefrontal cortex, and in many brain cancers (source GeneAtlas: http://geneatlas.roslin.ed.ac.uk/). The capability to modulate calcium homeostasis makes sorcin a possible player in brain functions and dysfunctions. Moreover, it is highly expressed in brain pathological conditions, e.g., in brains from Alzheimer’s disease (AD) patients vs. controls [37,38], in the frontal cortex of asymptomatic AD patients with respect to symptomatic AD patients [39], in amyloid plaques in sporadic vs. rapidly progressive AD patients, and in AD vs. cerebral amyloid angiopathy patients [40,41], thereby possibly protecting from acceleration progression that takes place in aggressive forms of the disease. Sorcin sequestration by aberrant forms of tau results in impaired calcium homeostasis and resistance to ER stress and may contribute to AD progression [31]. Sorcin is also overexpressed in frontal cortex tissues from frontotemporal dementia, with respect to control patients [42], in *substantia nigra* of Parkinson’s disease (PD) patients vs. controls [43], and in mitochondrial proteins from *substantia nigra pars compacta* pathologically verified PD patients vs. controls [44], is upregulated in MPP^+^-treated cells [36], and in induced pluripotent stem cells (iPSCs) derived from PD patients vs. control cells [45]. Sorcin is overexpressed in seven human and mouse models of Huntington’s disease, under the control of the ERSE-I (ER stress response element) promoter upstream sorcin gene, together with other proteins involved in ER stress and unfolded protein response [46]. 

The relevant role that sorcin seems to have in neurological processes and diseases, besides calcium homeostasis regulation, could also be due to the direct interaction with some key proteins, such as presenilin 2 (PS2), alpha-synuclein (AS), and the N-methyl-D-aspartate receptor. Sorcin directly interacts in a calcium-dependent fashion (in vitro, in cells and in human brain) with presenilin 2 (PS2) and alpha-synuclein (AS), which are important in AD and PD pathogenesis, respectively [47,48]; sorcin interacts with the C-terminal region of PS2, which is able to form low-conductance calcium channels in lipid bilayers [94], binds to RyR in a calcium-dependent way, and modulates calcium homeostasis [21]. 

Sorcin also interacts with the ionotropic glutamate receptor NMDAR1 subunit of the non-specific cation channel N-methyl-D-aspartate receptor in the caudate-putamen nucleus [95] and with annexins A7 and A11, that participate in the regulation of calcium homeostasis in astrocytes [96].

Sorcin is important for endometrium development and embryo implantation: it is downregulated in the mid-secretory (receptive) endometrium of women with unexplained infertility with respect to fertile women, and mediates endometrial angiogenesis, endothelial proliferation, migration, and invasion via regulation of the vascular endothelial growth factor (VEGF) pathway involving the vascular endothelial growth factor receptor 2 (VEGFR2), phosphatidylinositol 3-kinase (PI3K), Akt, and nitric oxide synthase (NOS) expression, possibly by regulating calcium homeostasis [76,77].

### 2.4. Sorcin in Cancer and Multidrug (MD)-resistant Tumors

MDR impairs the efficacy of chemotherapy against tumors, with over 90% treatment failure rate in metastatic cancers. Many mechanisms operate to confer drug resistance (Figure 1) [97]: scarce drug solubility and toxicity to normal tissues limit the doses of chemotherapeutic drugs that can be administered to cancer patients; pharmacokinetic issues, as absorption, distribution, metabolism, and elimination, reduce the amount of chemotherapeutic that effectively reaches cancer cells. Moreover, several mechanisms confer tumor cell drug resistance, e.g., low drug uptake caused by reduced expression or loss of influx transporters, enhanced drug efflux due to overexpression of drug efflux pumps, changes in lipid composition of the cell membrane, increased DNA damage repair, inhibition of apoptosis, alterations of cell cycle or checkpoints, off-target drug compartmentalization, increased drug catabolism, drug target structure modification, epithelial–mesenchymal transition (EMT).

Sorcin contributes to tumorigenesis and to the MDR phenotype via a series of mechanisms (Figure 1, Table 1).

Sorcin has been identified for the first time as a protein overexpressed in vincristine-resistant hamster lung cancer cells and denominated soluble, resistance-related, calcium-binding protein according to its main features [49]. Sorcin is expressed at high levels in many cancers, from many different tissues, usually with MD-resistant phenotype dependent on ABCB1 expression. The *SRI* gene is located in chromosome 7q21.12, in the same amplicon of ABCB1, the most important ATP-dependent efflux pump, capable of pumping a broad range of drugs and toxins out of cells [49]. Sorcin is “resistance-related” because its gene and *ABCB1* are often co-amplified in MD-resistant tumor cells [73]. For a long time, sorcin overexpression in MD-resistant cancer cells was considered as an accidental consequence of such genomic co-amplification [50]; on the contrary, in the last two decades, many studies have demonstrated that sorcin is an oncoprotein, and have revealed its role both as a marker and a cause of MDR. 

Sorcin is overexpressed in a number of cancers, such as lymphoma, leukemia (acute lymphoblastic, acute myeloid, chronic myeloid leukemias), myeloma, breast cancer, adenocarcinoma, gastric cancer, colorectal cancer, nasopharyngeal cancer, lung tumor, ovarian cancer, prostate cancer, tobacco-chewing mediated oral cancer, and particularly in MD-resistant tumors [20,24,25,26,34,50,51,52,53,54,55,56,57,58,59,60]; sorcin is overexpressed in glioblastoma, anaplastic astrocytoma, and oligodendroglioma, while is an important marker of poor clinical outcome in embryonal central nervous system tumors and a histological marker for malignant glioma [61,62,63,64,65]. According to the Human Protein Atlas (https://www.proteinatlas.org/ENSG00000075142-SRI), sorcin has moderate to strong cytoplasmic and nuclear positivity in most cancers, with the strongest staining displayed in low-grade gliomas, and is an unfavorable prognostic marker (*p* < 0.001) in pancreatic cancer (unfavorable), and an unfavorable quasi-marker (0.001 < *p* < 0.003) for liver cancer, cervical cancer, and endometrial cancer. However, sorcin is a favorable marker in lung cancer.

Sorcin transfection in different cancer cell lines, such as leukemia, lung, gastric, ovarian, and breast tumors, leads to increased drug resistance to chemotherapeutic drugs such as doxorubicin, vincristine, paclitaxel, etoposide, homoharringtonine, and 5-fluorouracil [24,25,34,60,66,74,98,99]. Conversely, sorcin silencing reverses MDR in leukemia, HeLa, breast, and colorectal cancer and nasopharyngeal carcinoma [24,26,52,54,60,66,67,68,69,70]. Human colorectal cancer cells express high amounts of sorcin, whose upregulation induces resistance to oxaliplatin, 5-fluorouracil, and irinotecan, while its downregulation sensitizes cells towards these drugs [26]. Sorcin is upregulated in many cisplatin-resistant cancers and tumor cell lines, such as leukemia, nasopharyngeal carcinoma, and lung cancer, while sorcin silencing increases cisplatin cytotoxicity and glutathione depletion [54,69,71]. Silencing of sorcin in MD-resistant myeloma cell lines increases cellular sensitivity to cisplatin and adriamycin, and decreases cell proliferation, cell cycle blockage, and apoptosis [32]. Moreover, Qu and collaborators showed that sorcin overexpression was associated with gemcitabine resistance and with poor prognosis in non-small cell lung tumor patients [56].

### 2.5. Sorcin Expression and ABCB1 Expression are Linked

Probably the expression of ABCB1 is the most significant and most characterized mechanism of MDR in which sorcin is involved.

Overexpression of many ATP-dependent efflux pumps, and especially ABCB1 (MDR1 or P-glycoprotein), is an important mechanism of resistance to a wide spectrum of chemotherapeutic drugs, including anthracyclines, taxanes, and Vinca alkaloids, in cancer cell lines and in many cancers, e.g., many solid and hematological tumors [100,101]. Sorcin overexpression increases ABCB1 expression, determining increased drug resistance to several drugs, while sorcin silencing decreases expression of ABCB1, increasing cell death, in gastric cancer cells, lung tumor cells, nasopharyngeal carcinoma, cervical carcinoma cells, and leukemias [24,32,34,52,57,60,66,68,69,74]. Sorcin increases ABCB1 expression by stimulating CREB1 phosphorylation by PKA, and binding of activated CREB1 to the cAMP response element (CRE) in the −716–−709 bp of the promoter of the *ABCB1* gene [34]. Sorcin silencing was also shown to inhibit ABCB1 by suppressing ERK and Akt [75]. 

Sorcin gene is in the same chromosomal region (7q21.12) and in the same amplicon of the ABC transporters *ABCB1* and *ABCB4*, both in human and mouse genomes (the rodent amplicon contains two ABCB1 variants, i.e., *ABCB1a* and *ABCB1b*). ABCB1 expression is increased upon treatment with chemotherapeutic drugs, and ABCB1 confers MDR when overexpressed or amplified [102,103,104,105,106,107,108,109,110,111,112]; further, genomic amplification, due to genomic instability or chromosomal rearrangements, is responsible for increased *ABCB1* gene copy number and/or transactivation of ABCB1 expression [113,114,115,116,117,118].

**Figure 5:**
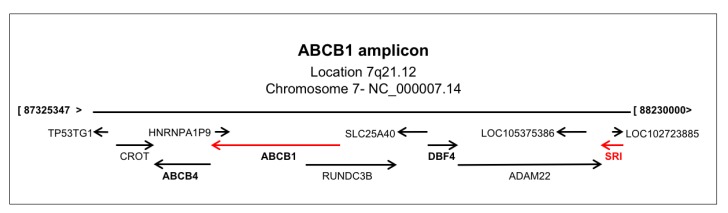
The *ABCB1* amplicon, located in chromosomal region 7q21, containing the sorcin(SOluble Resistance-related Calcium-binding proteIN) (*SRI*) gene.

Many studies report that a genomic amplification of the *ABCB1*-containing chromosomal region 7q21.12 occurs in MD-resistant cancers and that overexpression of genes of such region contributes to MDR (Figure 5) [51,73,119,120,121,122,123,124,125,126,127,128,129,130,131]. Amplification of the chromosomal region 7q21 containing *ABCB1* and *SRI* (sorcin gene) was described in multidrug-resistant leukemia, neuroblastoma, and lung tumor cells [122,125,132]. The amplicon includes the genes *SRI*, *ADAM22*, *DBF4*, *SLC25A40*, *RUNDC3B* (*RPIP9*), *ABCB1*, *ABCB4*, *CROT*, the *TP53TG1* long non-coding RNA, *TMEM243* and *DMTF1* (Figure 5), all of which were found to be associated with carcinogenesis and MDR. In particular, sorcin and DBF4 overexpression are drivers of MDR in several types of cancers; the development of inhibitors of sorcin expression and of CDC7-DBF4 activity as potential anti-tumor candidates was recently accomplished. DBF4 is a CDC7 kinase regulatory subunit, important in cell proliferation and DNA replication, overexpressed together with CDC7 in many primary tumors and cancer cell lines, such as diffuse large B-cell lymphoma, colorectal cancer, ovarian cancer, melanoma, breast cancer, and oral squamous cell carcinoma, and are markers of poor prognosis and of advanced tumor grade; many tumors contain extra copies of the *DBF4* gene [119,133,134,135,136,137,138]. Overexpression of CDC7-DBF4, reported in several human cancers, is considered a marker of MDR [123,126,133,139].

### 2.6. Sorcin, Metastatization, and EMT

Sorcin overexpression in gastric cancer tissue is related closely to the depth of invasion, staging severity of malignant tumors, and lymph node metastasis of gastric cancers [53].

Sorcin induces gastric tumor cell migration and invasion; sorcin silencing downregulates the expression of cathepsin Z, matrix metalloproteinases 2 and 9 (MMP2 and MMP9), and signal transducer and activator of transcription 3 (STAT3), resulting in the suppression of cancer growth and metastasis [78]. Sorcin overexpression facilitates cell migration, invasion, and epithelial–mesenchymal transition (EMT), which generates features associated with high-grade tumors, and leads to metastatic dissemination [67]. In colorectal HCT116 cells, sorcin activates EMT through activation of the PI3K/Akt/mTOR pathway [58]; in breast cancer, sorcin silencing inhibits metastatization and EMT, by increasing the expression of E-cadherin and decreasing that of vascular endothelial growth factor (VEGF) [67].

### 2.7. Sorcin Directly Binds Chemotherapeutic Drugs

**Figure 6:**
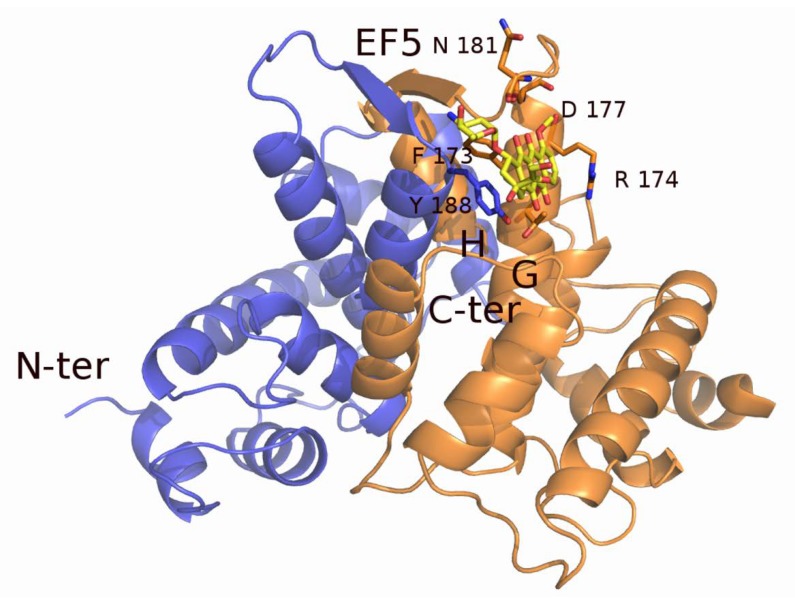
X-ray crystal structure of sorcin in complex with doxorubicin. The chemotherapeutic drug binds close to residues of the EF5 hand, interacting with residues of sorcin G- and H-helices. The two monomers of the sorcin dimer are colored blue and orange. The doxorubicin molecule (colored in yellow) and the residues interacting with it are represented as sticks.

Part of the MD-resistant phenotype can be attributed to the capacity of sorcin to directly bind chemotherapeutic drugs. In fact, sorcin binds with high-affinity doxorubicin, paclitaxel, vincristine, and cisplatin in vitro, as shown by experiments carried out with techniques such as Surface Plasmon Resonance, fluorescence titration, and X-ray diffraction [74]. Recently, a crystal structure of the sorcin–doxorubicin complex has been solved, allowing the identification of one of the two binding sites for doxorubicin, close to the interface of the two sorcin monomers. The doxorubicin molecule is placed between the EF5 loop, the G helix, and the EF4 loop, and is involved in interactions with residues Phe173, Arg174, Asp177, and Tyr188 of the second monomer (Figure 6) [74]. Upon doxorubicin treatment, sorcin cellular localization changes, indicating a possible interaction also inside the cell. These findings show that sorcin can limit the toxic effects of doxorubicin (and possibly also of other drugs) in the cell, acting as a drug scavenger [74].

### 2.8. Targeting Sorcin

The involvement of sorcin in MDR has been shown for several cancer cell lines and, although it likely occurs through the several mechanisms described, the general inference is that lowering sorcin expression should have the effect to revert MDR. This body of results shows that sorcin can be considered a novel potential anticancer drug target. Studies aimed at targeting sorcin have been recently carried out, using different approaches.

MicroRNAs are negative gene regulators: miR-1, targeting the 3’-UTR region of sorcin gene *SRI* in its position 29–35, modulates calcium transients in cultured cardiomyocytes, and its expression is downregulated in human heart failure specimen and murine models [140]. In MD-resistant gastric cancer cells, miR-1 is highly downregulated; miR-1 overexpression increases apoptosis and promotes doxorubicin and vincristine accumulation in tumor cells, by acting on sorcin expression [98]. Sorcin overexpression partially reverses the effect of miR-1 in MD-resistant gastric tumor cells [98]. miR-1 may, therefore, be used as a therapeutic molecule vs. sorcin-dependent MDR. Off-target effects of miR-1, however, are possible, since the molecule is known to regulate other targets, such as HSP60, a component of the defense mechanism against diabetic myocardial injury, and the ets1 proto-oncogene, which plays a fundamental role in the extracellular matrix degradation [141,142].

Dihydromyricetin (DMY), a dihydroflavonol compound with anti-oxidant, anti-inflammatory, anti-bacterial, and anti-tumor effects, is able to reverse MDR in adriamycin-dependent MD-resistant breast cancer and leukemia cell lines and in a nude mice model, and to increase adriamycin cytotoxicity, by decreasing sorcin expression (both mRNA and protein), and consequently ABCB1 levels, via ERK/Akt pathways [24,75]. DMY increases intracellular free Ca^2+^ concentration, reactive oxygen species (ROS) levels, and expression of caspase 12, i.e., markers of ER stress-linked apoptosis, and regulates expression of markers of mitochondrial apoptosis, such as caspase 9, caspase 3, Bcl-2, Bax, and PARP [24]. Ondansetron (OND), an antiemetic drug used during tumor chemotherapy, also has a reversal effect in MDR due to sorcin expression, especially when used in combination with DMY, and restores P53 function by suppressing MDM2/MDMX, thus determining G2/M arrest and apoptosis; both DMY and OND may act by binding sorcin with high affinity [75]. It is possible that other targets may be regulated via the same ERK/Akt pathway, though the effect of DMY seems rather specific and dependent on binding to sorcin.

Haishengsu (HSS), a protein extract from the seashell *Tegillarca granosa*, promotes apoptosis in adriamycin-resistant leukemia cells inoculated in mice, by reducing expression of sorcin and ABCB1 [143] (other effects on other targets cannot be excluded, considering that this protein extract is poorly characterized from a molecular viewpoint). Administered to patients with acute leukemia in combination with different chemotherapy regimens, HSS increased treatment efficacy (in particular remission rate) and improved quality of life (decreasing nausea and vomiting) [144].

Some small compounds have activity on sorcin expression, among others. Calcitriol, the active form of Vitamin D, binds to the calcitriol receptor, also called vitamin D receptor or VDR. The VDR-calcitriol complex migrates to the nucleus where it acts as a transcription factor activating metabolic pathways with multifaceted effects on calcium homeostasis, leading to differentiation and anti-proliferative action and regulating sorcin expression, mostly increasing the 19-kDa form of the protein [145].

Palmitate, a free fatty acid highly circulating in obese children, induces sorcin downregulation [146], and subsequent increases in glucose-6-phosphatase catalytic subunit-2 levels contribute to lipotoxicity, to ER calcium depletion, and to ER stress in pancreatic β-cells [23]. The addition of metformin during 2-day palmitate exposure normalized oxygen consumption rate and sorcin levels [147].

Triptolide is a diterpenoid epoxide which is produced by the thunder god vine, *Tripterygium wilfordii*. It has antitumor activity in both non-resistant ovarian cancer SKOV3 and cisplatin-resistant SKOV3/DDP cells, likely through inducing apoptosis and regulating MMP-2, sorcin, and vascular endothelial growth factor expression [148].

Of course, given the high expression of sorcin in normal tissues (especially heart and brain) and its importance in regulating many important cellular events, targeting sorcin in cancer without undesirable off-target toxicity is not easy. This is a problem often encountered with many chemotherapeutic drugs: off-target activity, undesired toxicity vs. normal non-tumor cells, and development of resistance to drugs are the most important problems in anti-cancer treatments. Many approaches have been recently used to widen the therapeutic window of such molecules, and the use of antibodies and antibody-drug conjugates targeting cancer cells, or of genetically modifying techniques is now a reality.

To overcome off-targeting sorcin, we expect that the most useful approaches will be either via specifically targeting sorcin expression or its interaction with other proteins. A deep characterization of sorcin interactome is required to be able to aim at specific interaction and reduce off-target effects. Structural studies performed so far indicate that two hydrophobic regions become exposed upon calcium binding to EF1 and EF3 (Figure 3). Since, at least in principle, the two surfaces could mediate interaction with different partners, targeting different sites on the sorcin surface could allow specific modulation of interaction, resulting in a focused action.

## 3. Conclusions

In conclusion, sorcin represents an intriguing cancer target, due to its co-amplification with xenobiotic efflux pumps and to its role in cellular calcium signaling and calcium homeostasis. Many efforts have been spent in the dissection of the mechanisms of sorcin action in diverse pathophysiological settings, paving the way towards the development of successful and novel therapeutic strategies. We think that studies on the recently obtained sorcin^−/−^ knock out mouse [18,28] will give important information on the roles of sorcin not only in cancer and in MDR, but also in brain and muscle development, in lipid metabolism, in diabetes, and in neurodegenerative diseases.

## Figures and Tables

**Table 1:** Sorcin: Roles in cells, tumors, and multidrug resistance (MDR).

Events:	Sorcin Role:	References Relevant Studies
Calcium homeostasis	Regulation of Ca^2+^ channels, pumps, exchangers	[4,5,6,7,8,9,10,11,12,13,14,15,16,17,18,19,20,21]
	Regulation of ER and cytosolic Ca^2+^ concentration	[4,6,7,8,9,10,11,12,13,14,15,16,17,18,19,20,21,22,23,24,25,26,27,28]
	Regulation of heart-muscle contraction	[6,7,8,9,10,11,12,13,14,15,16,17,18,29,30]
Cellular metabolism	ER stress sensor, unfolded protein response regulation	[20,22,26,27,28,31]
	Regulation of mitosis, cytokinesis, cell cycle	[17,22,26,27,32]
	Regulation of kinases	[22,30,33,34]
	Regulation of glucose metabolism	[28,35]
Neurodegeneration	Overexpression in neurodegenerative diseases	[31,36,37,38,39,40,41,42,43,44,45,46,47,48]
Cancer	Overexpression in tumors	[20,23,24,25,26,34,49,50,51,52,53,54,55,56,57,58,59,60,61,62,63,64,65]
	Increase of MDR	[24,25,26,32,34,52,54,56,60,66,67,68,69,70,71]
Cell death	Regulation of cell death	[9,15,25,42,43,44,45,46,47,48]
Drug elimination	Drug binding-elimination	[72]
Efflux pumps	Increased expression of ABCB1	[24,32,34,52,57,60,66,68,69,73,74,75]
EMT	Increase of EMT	[58,67]
Angiogenesis	Increase of invasion and angiogenesis	[67,76,77]
Metastatization	Increase of metastatization	[53,67,78]
